# General Strategy to Prepare Single-Layered Ag–Au–Pt Nanocrystal Ternary-Coated Biomass Textiles through Polymer-Driven Self-Assembly

**DOI:** 10.3390/nano10030495

**Published:** 2020-03-10

**Authors:** Liheng Gao, Jundan Feng, Sijun Xu, Min Shi, Lirong Yao, Lu Wang, Zhongtian Yang

**Affiliations:** 1 National & Local Joint Engineering Research Center of Technical Fiber Composites for Safety and Protection, School of Textile and Clothing, Nantong University, Nantong 226019, China; 2 Key Laboratory of Textile Science and Technology, Ministry of Education, College of Textiles, Donghua University, Shanghai 201620, China

**Keywords:** assembly, textiles, nanostructures, clean production

## Abstract

Current metal nanomaterials for developing nanofunctional textiles are mostly based on metal nanoparticles (NPs) that show aqueous instability, a tendency to aggregate, and low chemical affinity to biomass textiles, leading to low nano-metal uptake during finishing, significant declines in function, and nano-pollution. Herein, we demonstrate a strategy to transform metal (Ag, Au, and Pt) NPs into homogenous hyperbranched poly(amide-amine) (HBPAA)-encapsulated NPs showing high water solubility, oxidative resistance, and affinity to biomass materials upon surface capping with HBPAA. The proposed method represents a universal, simple, clean, and efficient self-assembly technology to produce monolayered Ag–Au–Pt ternary-coated biomass textiles. The combination of Ag, Au, and Pt NPs yields a positive potential of approximately +37.12 mV depending on the metal concentration and could simultaneously self-assemble onto natural fibers, including cotton, silk, and wool, through the one-step impregnation of textiles. Increasing the temperature and concentration of the mixture favors the self-assembly process. A mixture of 30–110 mg/L Ag, Au, and Pt NPs could nearly completely anchor onto cotton, silk, and wool textiles after impregnation at 100 °C for 1 h without chemical assistance, thereby indicating the possibility of clean production. As-prepared functional cotton, silk, and wool possessed similarly high antibacterial activities, and a mixture containing over 1500 mg/g NPs inhibited 99% of the *Escherichia coli* and *Staphylococcus aureus* in the sample textiles. The developed coating technology is simple, clean, controllable, and broadly applicable; thus, it could be potentially applied in functional textiles.

## 1. Introduction

Interest in nanomaterials, especially nanoparticles (NPs), as a basic building block in advanced materials has grown on account of their unique size-dependent physical, optical, electronic, and chemical properties [1]. The unique (nano) size of NPs produces an obvious quantum confinement effect, which is defined as an increase in bandgap accompanied by the quantization of energy levels to discrete values [2]. NPs possess ultrahigh surface-to-volume ratios and nontrivial surface areas. The atom ratio of surface/volume of a 20 nm NP is approximately 10%. However, when the NP diameter is decreased to 2 nm, the surface atoms comprise up to 80% of the total. Typical quantum confined properties are manifested by the size-dependent photoluminescence of nanocrystalline semiconductor quantum dots, plasmonic resonances of metal NPs, electrical properties of carbon nanomaterials, and paramagnetism and catalytic properties of certain inorganic NPs [3]. When the size of metal NPs (e.g., Ag, Au, and Pt) is smaller than the wavelength of light, free electrons can be displaced from the lattice of positive ions and collectively oscillate; this phenomenon is called localized surface plasmon resonance [4]. Moreover, metal nanomaterials have strong catalytic capacity due to their large specific surface area and surface chemical activity [5,6]. Ag, Au, and Pt NPs are important nanocatalysts that are used in many catalytic reactions for hydrogen purification in fuel cells, chemical synthesis, and electrocatalysis to control environmental pollution [7]. Metal NPs also have broad-spectrum antimicrobial effects due to their ultrahigh specific surface area [8].

The integration of metal NPs with regular-sized materials, such as biomass textiles, has become a popular research direction, especially for wearable and functional textiles [9,10,11]. However, although various preparation strategies, such as dipping coating, spraying, In-Situ reduction, and sputtering, have been developed, the preparation of a uniformly monodispersed multi-metallic coating on biomass fibers (e.g., silk fibers) through a simple, versatile, and environment-friendly manner continues to challenge scholars [12]. Polymer-functionalized inorganic nanomaterials consist of an inorganic core made up of atoms with numbers ranging from several hundreds to a few thousands and a polymer outer layer. This fascinating structure imparts metallic NPs with designable physicochemical properties, including solubility, surface potential, surface functional groups, and surface chemical affinity [13,14,15].

We infer that different metal NPs capped with the same functional polymer may inherit the chemical characteristics of the latter. Water-soluble polymer-capped heterogeneous metal NPs can be regarded as a type of organic NPs, i.e., polymer NPs, regardless of the composition of their nano-metal core. Such polymer NPs may possess a chemical affinity identical to that of natural polymers, such as cellulose and protein, compared with their independent NPs, thus suggesting the possibility of preparing a uniformly monodispersed nanohybrid coating with the help of electrostatic repulsion. However, three preconditions should be met when selecting a capping polymer, which determines the final physicochemical functions of NPs: solution stability, surface potential, and surface chemical activity. A capping polymer should have good protective ability toward NPs and prevent NPs from undergoing unordered crystal growth and oxidation [16]. Capping polymers with strong intra/intermolecular bonding interactions should be avoided because these forces increase the viscosity of the colloidal solution system and decrease the degree of freedom of NPs. Finally, an ideal capping polymer should possess a strong surface potential and as many unbonded terminal functional groups as possible to protect the NPs from aggregation and endow them with chemical affinity to target polymers [15,17,18]. Therefore, micromolecules and linear polymers are unsatisfactory candidates for the capping of metal NPs.

In the present study, hyperbranched polymer reductive amino-terminated hyperbranched poly(amide-amine) (HBPAA) was chosen to encapsulate various metal NPs because of its three-dimensional cavity structure, ultralow relative viscosity, and high density of positively charged amino-terminated functional groups; these properties provide NPs with high charge and steric hindrance and electrostatic attraction and hydrogen-bonding interactions to most biopolymers [19]. Moreover, AgNO_3_, HAuCl_4_, and H_2_PtCI_6_ are reduced to metallic-state nanocrystals in boiled water and in situ capped by reductive HBPAA to create three colloidal solutions of HBPAA-capped Ag, Au, and Pt NPs. Then, these solutions are transformed into a homogeneous cationic dye possessing chemical affinity toward biomass materials, including cotton, silk, and wool. The mixture of colloidal Ag, Au, and Pt NPs has high solution stability and strong self-assembly ability toward natural cotton, silk, and wool textiles, similar to their discrete NPs. In addition, the mixture can spontaneously and nearly completely monodispersed on the three biopolymer textiles driven by long-range electrostatic attraction and short-range hydrogen-bonding interactions between positively charged NPs and negatively charged hydroxyl- and/or amide-containing cellulose and proteins. Electrostatic repulsion can prevent the agglomeration and stacking of NPs on fiber surfaces, and the proposed technology can efficiently assemble NPs onto biomass surfaces in a simple, clean, and monodispersed manner.

## 2. Experiment

### 2.1. Materials

Cotton, silk, and wool textiles were obtained from Zhangjiagang Nellnano Technology Co., Ltd. (Suzhou, China). HBPAA was synthesized as described in our previous paper [20,21]. AgNO_3_, HAuCl_4_·4H_2_O, H_2_PtCl_6_·6H_2_O, NaBH_4_, NaOH, and HNO_3_ were purchased from Sinopharm Group Chemical Reagent Co. Ltd. (Shanghai, China). *Staphylococcus aureus* (ATCC 6538) and *Escherichia coli* (ATCC 8099) were purchased from China General Microbiological Culture Collection Center (Beijing, China). Nutrient broth and nutrient agar were purchased from Scas Ecoscience Technology Inc. (Shanghai, China).

### 2.2. Preparation of Single-Layered Ag–Au–Pt Nanocrystal Ternary-Coated Biomass Textiles

HBPAA-capped Ag and Au NPs were prepared in accordance with our previously reported method [20,21]. HBPAA-capped Pt NPs were prepared by slowly adding 1.3 g/L sodium borohydride solution to a boiled mixed solution of chloroplatinic acid and HBPAA (pure Pt content: 500 mg/L; HBPAA content: 2.5 g/L). After reaction for 30 min, the resultant solution was dialyzed to remove remnant small-molecule compounds.

Exactly 100 mL of 10–200 mg/L solutions of Ag, Au, and Pt NPs was mixed together and magnetically stirred for 30 min; here, the concentration ratio of pure solutions of Ag, Au, and Pt NPs was set to 1:1:1. Then, 1 g of textiles was impregnated in 50 mL of the mixed solution and held for 0–1 h at temperatures ranging from 30 to 100 °C. Finally, the resulting textile samples were collected, rinsed with deionized water, cured in an oven at 120 °C, and stored in a dark place. The residual concentrations of Ag, Au, and Pt NPs in the solution (C_Ag_, C_Au_, C_Pt_; mg/L) were measured by inductively coupled plasma atomic emission spectroscopy (ICP-MS). The NP mixture content (*C*, mg/kg) of the treated textiles was calculated by the following formula:

(1) $$C=50[C_{0}-(C_{\mathrm{Ag}}+C_{\mathrm{Au}}+C_{\mathrm{Pt}})]$$

where *C* is the total content of Ag, Au, and Pt NPs in the textiles; *C*_o_ is the initial total concentration of Ag, Au, and Pt NPs; and C_Ag_, C_Au_, and C_Pt_ are the respective concentrations of Ag, Au, and Pt NPs in the residual solution.

### 2.3. Measurements

The morphological structure of the metal NPs was obtained by using a transmission electron microscope (TEM; JEOL 2100F, Tokyo, Japan) at a beam acceleration voltage of 200 kV. The surface structure and morphology and chemical composition of the fabrics were characterized by a field-emission scanning electron microscope (SEM; Zeiss Gemini FESEM 300, Oberkochen, Germany) equipped with an energy-dispersive X-ray spectroscope (EDS; Oxford X-Max 80, Oxford, UK) at an acceleration voltage of 15 kV. The crystal structures of the samples were identified by using an X-ray diffractometer (XRD; Shimata XD-D1, Kyoto, Japan). The elemental concentration of the solution was measured by ICP-MS (PerkinElmer NexION 350, Waltham, MA, USA).

The antibacterial activity of the Ag, Au, and Pt NP-coated natural textiles (cotton, silk, and wool) was assessed according to the standard shake-flask method. *E. coli* (ATCC 8099) and *S. aureus* (ATCC 6538) were chosen as test strains, cultured in lysogeny broth (LB) medium at 37 °C, shaken at 170 rpm for 12 h, and then diluted to 2–4 × 10^4^ colony forming units (CFU)/mL in 0.1 M of phosphate-buffered saline (monopotassium phosphate, pH 7.0). After washing with 75% ethanol and dried in an oven, all textile samples were cut into 2 × 2 cm^2^ pieces, dipped into 4 mL of the bacterial solutions, and shaken at 120 rpm on a rotary shaker at 37 °C for 15 h. Finally, 100 μL of the bacterial suspension was taken, serially diluted with saline (×10^1^, ×10^2^, ×10^3^), transferred onto LB agar, and then incubated at 37 °C for 24 h. The percentage reduction (cfu, %) was determined as follows:

(2) $$\mathrm{cfu}\left(\%\right)=\frac{C-A}{C}\times 100$$

where *C* and *A* are the bacterial colonies of the blank and treated textiles, respectively. All antibacterial experiments were performed in triplicate with three substrates, and mean values were calculated. Differences among groups were analyzed with one-way ANOVA followed by the Least-Significant Difference (LSD) test using SPSS20.0. *p* < 0.05 was considered a significant difference.

## 3. Results and Discussion

**Figure 1:**
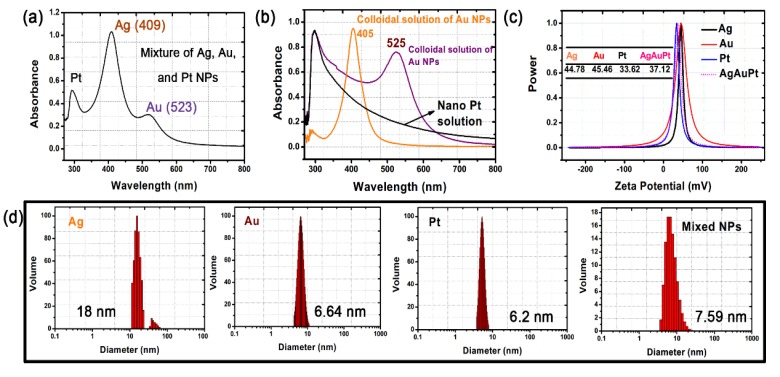
(**a**,**b**) UV–vis spectra, (**c**) zeta potentials, and (**d**) size distributions of the single and mixed solutions of Ag, Au, and Pt nanoparticles (NPs).

Core–shell structured nanomaterials integrating different nano-components not only inherit the different functions of their separate parts but also produce new functions through synergistic effects. Surface coating metal NPs with functional polymers is important for practical applications because the strategy yields improved water solubility, inoxidizability, dimensional stability, chemical affinity to organic material surfaces, and even biosafety and biodegradability. In the present study, three colloidal solutions of positively charged HBPAA-capped Ag, Au, and Pt NPs were prepared and mixed to obtain homogenous colloidal NPs (Figure 1a). The mixed NPs showed similar surface physicochemical characteristics, including shapes and sizes, surface potentials, and surface functional groups, and high affinity toward biomass fibers due to their identical HBPAA coat. Mixed NPs in solution can proactively recognize and bind to the surface of hydroxyl and carboxyl biopolymers due to the molecular binding forces of HBPPA and form a monodispersed NP coating. Macroscopic fibers and the related textiles may gain various combined functions, such as antimicrobial, medical imaging, and air purification properties, when integrated with Ag, Au, and Pt NPs.

**Figure 2:**
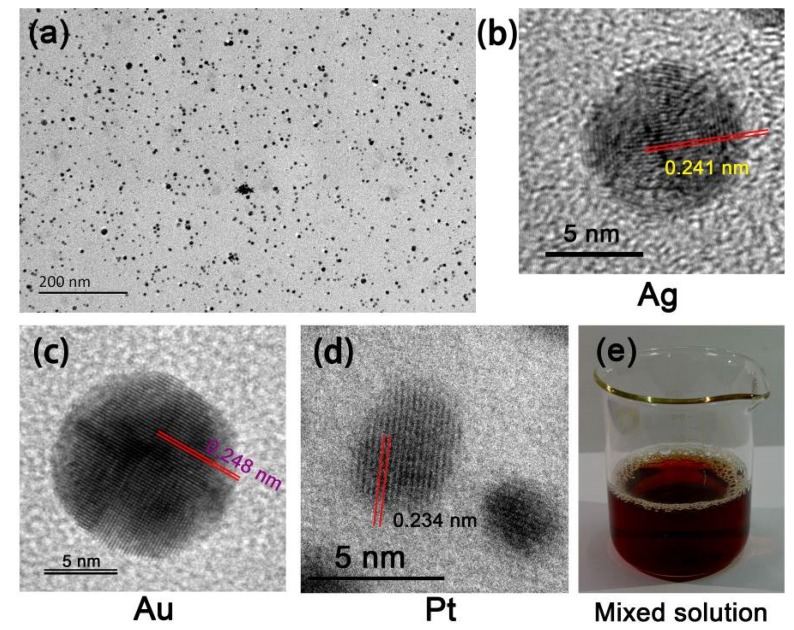
(**a**) TEM and high-resolution TEM photographs of the (**b**) Ag, (**c**) Au, and (**d**) Pt NPs. (**e**) Photograph of the mixed solution.

The HBPAA-capped Ag, Au, and Pt NPs revealed similar surface potentials and good compatibility in water due to strong electrostatic repulsion forces among NPs. The zeta potentials of the pure Ag, Au, and Pt NPs in water were +44.78, +45.46, and +33.62 mV, respectively (Figure 1c) [22]. The mixed NPs possessed a surface potential of +37.12 mV, indicating no charge interference or cancellation among the three NPs. The UV–vis spectra of the mixed solution showed two typical peaks at approximately 409 and 523 nm and a shoulder peak at 294–329 nm, which can be assigned to Ag, Au, and Pt NPs, respectively, and confirm the water miscibility of the NPs (Figure 1a,b). TEM images of the Ag, Au, and Pt NPs showed numerous NPs monodispersed on the Cu support due to electrostatic repulsion (Figure 2). The Ag, Au, and Pt NPs revealed similar particle sizes in the range of 5–60 nm (Figure 1d and Figure 2b–d). Specifically, the Ag and Au NPs revealed a regular spherical structure with average particle sizes of 18 nm and 6.64 nm, while the Pt NPs were mostly oval and had a little smaller diameter of approximately 6.2 nm. Free Ag, Au, and Pt NPs in a mixed solution are an important precondition for the subsequent self-assembly process.

**Figure 3:**
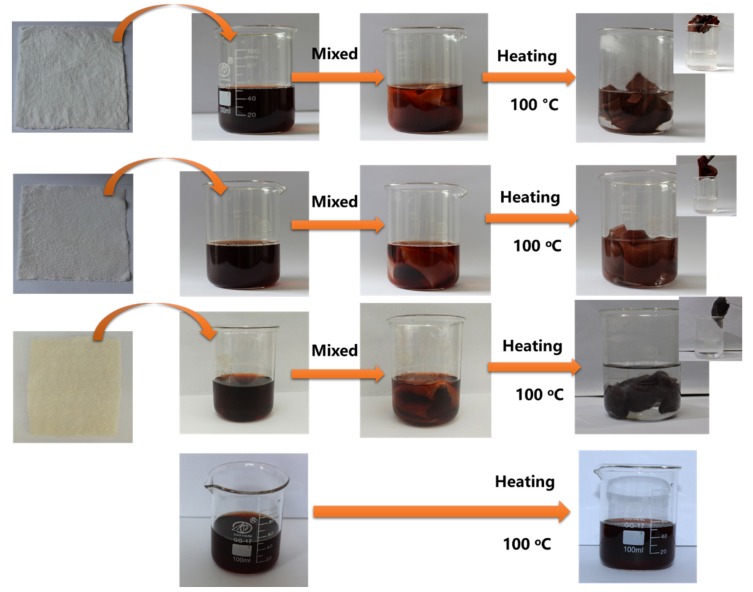
Schematic of the heat-induced self-assembly of mixed Ag, Au, and Pt NPs on cotton, silk, and wool textiles (110 mg/L).

Cotton, silk, and wool are chemically composed of negatively charged cellulose and proteins that can form various ingenious flexible and porous fiber structures. Dependent on their unique physicochemical structure, cationic HBPAA showed strong affinity to negatively charged biomass fibers [23]. The self-assembly process is shown in Figure 3. The mixed solution quickly faded and became colorless within 3 min, while the cotton and silk textiles turned dark brown after hydrothermal treatment, thus indicating that the Ag, Au, and Pt NPs were nearly completely adsorbed to fiber surfaces [24]. However, compared with cotton and silk, the treated wool showed a much darker brownish red color probably because of the light-scattering effect of wool scales.

**Scheme 1:**
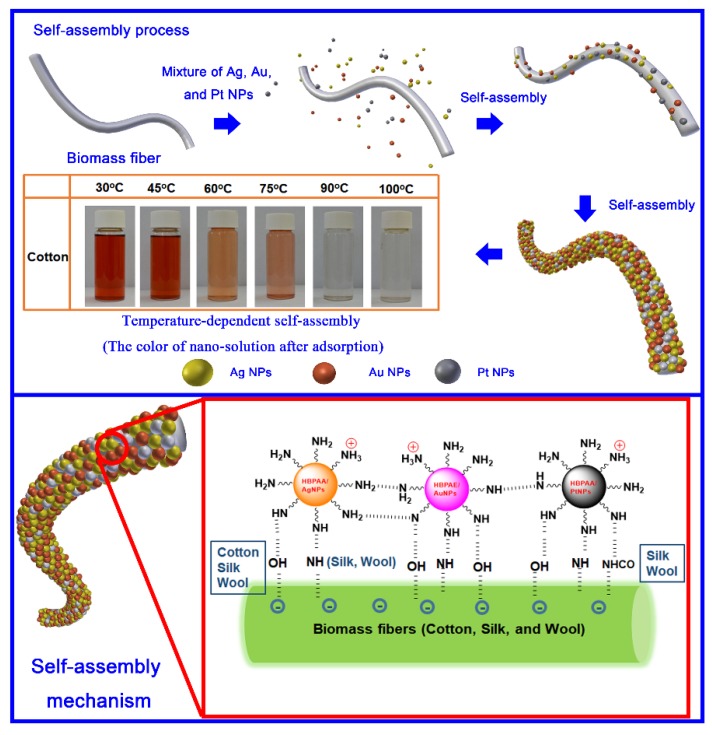
Possible mechanism of self-assembly of homogenous hyperbranched poly(amide-amine) (HBPAA)-capped NPs on biomass fibers.

Mixed Ag, Au, and Pt NPs encapsulated within the nanocellular HBPAA could be regarded as homogeneous HBPAA NPs. Therefore, the self-assembly of NPs on textiles could be attributed to the physicochemical interactions between HBPAA and the biomass fibers (Scheme 1). Cotton, silk, and wool are naturally composed of hydroxyl-abundant cellulose and acylamino-containing protein macromolecules. Cationic spherical HBPAA, which features numerous active terminated amino groups, shows high chemical compatibility with negatively charged hydroxyl and acylamino polymers in the water phase. Furthermore, the sponge-like amorphous regions of natural fibers can spontaneously adsorb NPs into their bodies, thereby forming an embedded structure. In summary, the long-range electrostatic, short-range hydrogen-bonding, and capillary forces were responsible for the self-assembly of HBPAA-capped Ag, Au, and Pt NPs on biomass textiles. 

**Figure 4:**
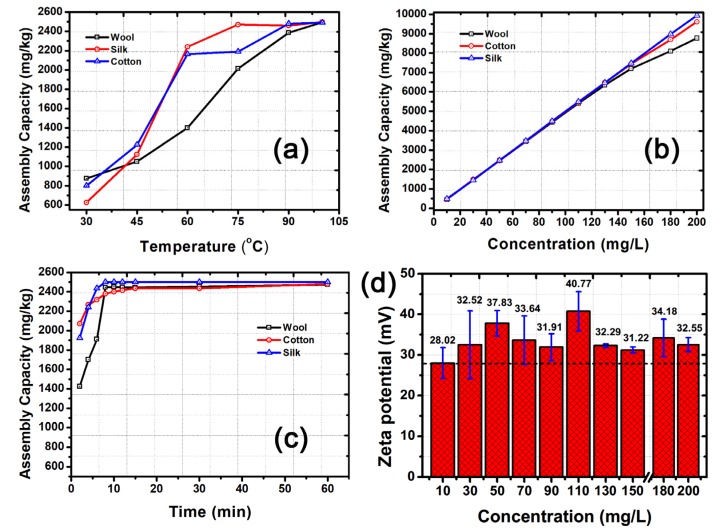
(**a**) Temperature-, (**b**) concentration-, and (**c**) time-dependent self-assembly capacity of mixed Ag, Au, and Pt NPs on cotton, silk, and wool textiles in solution. (**d**) Changes in the zeta potential of the mixed solutions as a function of NP concentration. The concentration of mixed NPs in Figure 4a,c was 50 mg/L. The solution temperature in Figure 4b was 100 °C.

The assembly capacity of the mixed Ag, Au, and Pt NPs on cotton, silk, and wool fibers showed similar laws (Figure 4). Increases in temperature can greatly promote the chemical activity of HBPAA and increase the capacity of the NPs for self-assembly on the fibers, especially at >75 °C (Figure 4a). Wool is less sensitive to temperature compared with cotton and silk but showed a higher uptake of NPs at low temperature (30 °C). By contrast, the self-assembly of NPs on cotton and silk was more temperature-dependent. The assembly capacity of NPs increased from 800 and 625 mg/kg to 2240 and 2165 mg/kg and then to 2460 and 2480 mg/kg, respectively, as the temperature increased from 30 to 60 °C and then to 90 °C. The corresponding uptake efficiency of mixed NPs on cotton and silk reached up to 98.4% and 99.2% at the temperature of 90 °C (Figure 4a). Such increase is due to the dramatic expansion of fibers in high-temperature aqueous solution, which leads to a sharp increase in porosity in the amorphous regions of the fibers [25,26,27]. With the temperature set to 100 °C, the self-assembly capacities of the mixed NPs on three textiles to textiles depended on the concentration of NPs (Figure 4b). The capacities of NPs to assemble on cotton, silk, and wool reached 9615, 9940, and 8775 mg/kg, respectively, as their concentration increased to 200 mg/L. Whereas mixed NPs at concentrations between 30 and 110 mg/L showed over 99% uptake efficiency, NPs at concentrations from 130 to 200 mg/L showed a gradual decrease in uptake efficiency from 99.7%, 99.5%, and 97.7% to 96.1%, 99.4%, and 87.8% for cotton, silk, and wool, respectively. Such results could be attributed to the increased charge repulsion and decreased free energy of positively charged HBPAA-capped NPs, which gradually counteract the electrostatic attraction between NPs and the fibers. In particular, the self-assembly efficiencies of 10 mg/L nanomaterials for cotton, silk, and wool textiles were 95.4%, 96.2%, and 97.8%, respectively, which is slightly lower than the efficiencies observed at 30–110 mg/L. The 10 mg/L mixed NPs had a zeta potential of only +28.02 mV (Figure 4d). This can explain the weakened uptaking efficiency of NPs in textile materials. The self-assembly rate of metal NPs in the textiles initially high but decreased remarkably 12 min later (Figure 4c). Mixed NPs nearly completely adsorbed to textiles in 60 min, thereby indicating their high self-assembly efficiency.

**Figure 5:**
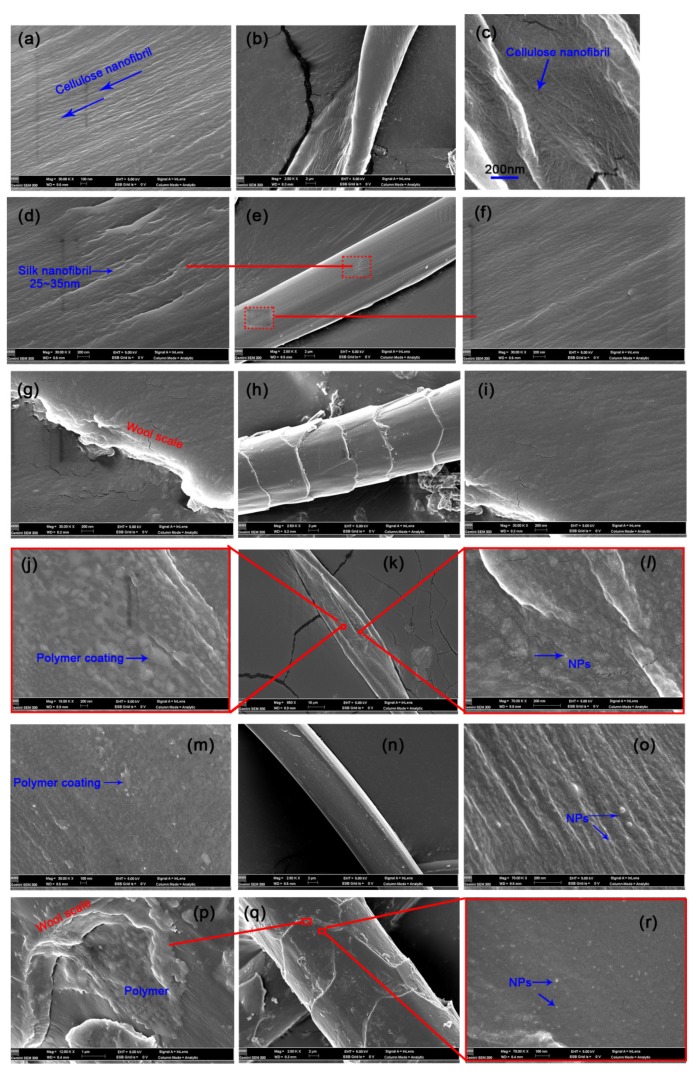
FESEM analysis of (**a**–**c**) pure cotton, (**d**–**f**) silk, (**g**–**i**) and wool and Ag, Au, and Pt NP ternary-coated (**j**–**l**) cotton, (**m**–**o**) silk, and (**p**–**r**) wool (NP content: approximately 5500 mg/g).

The surface morphology of the textiles is shown in Figure 5. Cotton, which comes from seed hair, is the most widely used among natural cellulosic fibers. The longitudinal view of cotton shows a ribbon-like shape with convolutions (twist) at irregular intervals and a clean surface possessing numerous parallel or netted arranged cellulose nanofibrils (Figure 5a–c) [28]. Unlike cotton, silk consists of high-purity biological protein, which is similar to the structure of synthetic fibers, such as nylon. Mulberry silk, a typical solid fiber extruded and spun by silkworms in the form of a cocoon, has an elegant and lustrous appearance due to its triangular cross-section and smooth surface (Figure 5d–f) [29]. Wool fibers are another type of protein fibers that are widely used to provide warmth and good resiliency. The longitudinal view of wool fibers shows characteristic overlapping scales pointed toward the tips of the fibers (Figure 5g,i). Compared with the ordered nanofibrils on the silk surface, nanofibrils on the wool surface are uneven, darker, and glossier. The three textiles showed remarkably different surface characteristics compared with their original forms after integration with mixed HBPAA-encapsulated NPs. As shown in Figure 5j–r, the HBPAA coatings presented in the form of polymer nanofilms (bright white films on Figure 5j,m,p). On the contrary, spherical HBPAA-encapsulated NPs were found to monodisperse evenly on the cotton and silk surfaces (Figure 5l,o). Unlike the loose deposition, when NPs are applied to textiles using traditional physical impregnation or spraying strategies, tight films strongly glued to cotton and silk were noted when the NPs were combined with HBPAA (Figure 5l,o,r). Compared with that on cotton and silk, free HBPAA adhered to the junction of wool scales, which greatly increased the microscopic roughness of wool after coating with HBPAA (Figure 5p). This finding may explain the darker color of Ag, Au, and Pt NP-coated wool textiles compared with those of cotton and silk. Free HBPAA did not interfere with the monodistribution characteristics of HBPAA-capped NPs on the wool surface, which is attributed to strong electrostatic repulsion among mixed NPs (Figure 1c and Figure 5r). In summary, HBPAA-encapsulated Ag, Au, and Pt NPs showed the same surface chemical properties, i.e., high positive surface potentials and high affinity to cellulose and protein. The good monodispersion of the mixed NP coatings was due to the synergistic effects of the electrostatic attraction and hydrogen-bonding interactions between NPs and the biomass fibers and electrostatic repulsion among Ag, Au, and Pt NPs.

**Figure 6:**
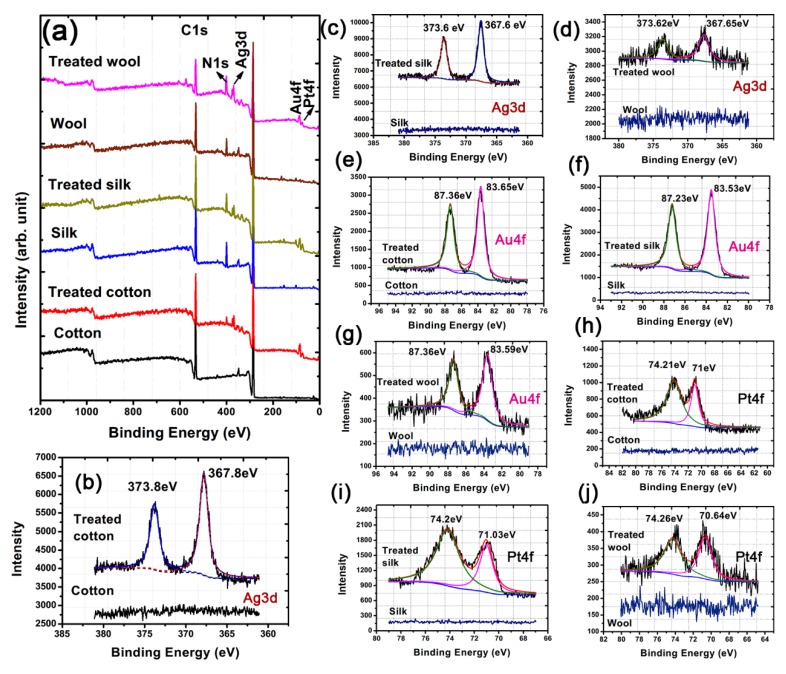
(**a**) Wide-scan, (**b**–**d**) Ag 3d, (**e**–**g**) Au 4f, and (**h**–**j**) Pt 4f XPS spectra of blank and treated cotton, silk, and wool textiles (NP content: approximately 5500 mg/g).

The amino functional group-driven self-assembly of free HBPAA and HBPAA-capped Ag, Au, and Pt NPs on biomass fibers indicates a remarkable surface chemical transformation, which can be evidenced by XPS (Figure 6) [30]. Typical characteristic peaks that could be assigned to C 1s and O 1s for cotton and C 1s, N 1s, and O 1s for silk and wool were found at approximately 284, 398, and 532 eV in the wide-scan XPS spectra. Such signals were mainly derived from cellulose or protein (Figure 6a). However, additional Ag 3d, Au 4f, and Pt 4f peaks were detected at approximately 368, 84, and 71 eV for all treated textiles, thus confirming the attachment of Ag, Au, and Pt NPs in the three fibers. The direct evidence for adhesion of HBPAA on biomass fibers can be found in cotton. Comparison of the wide-scan XPS spectra of treated and pure cotton reveals an additional N 1s peak in the former, thus suggesting the existence of amine-terminated HBPAA (Figure 6a).

Metallic NPs, particularly Ag NPs, are prone to oxidation in an oxygen-rich air environment when they are transferred from water to the fiber surface. Therefore, capping of the three NPs with reductive 3D HBPAA has other important benefits, i.e., capping isolates the NPs from one another and gaseous O_2_, thereby enhancing their physical and chemical stability [16]. The deconvoluted fitting results of Ag 3d indicate peak positions that are nearly identical to those of standard metallic Ag, Au, and Pt for cotton, silk, and wool textiles (Figure 6b–j and Table 1). In addition, differences between the 3d_5/2_ and 3d_3/2_ peaks of Ag (approximately 6.0 eV) and between the 4f_5/2_ and 4f_7/2_ peaks of Au (approximately 3.7 eV) and Pt (approximately 3.33 eV) are very close to the standard values of Ag^0^ (6.0 eV), Au^0^ (3.65 eV), and Pt^0^ (3.35 eV) [31,32]. This observation suggests that the majority of the Ag, Au, and NPs in the treated samples are in the metallic state.

**Figure 7:**
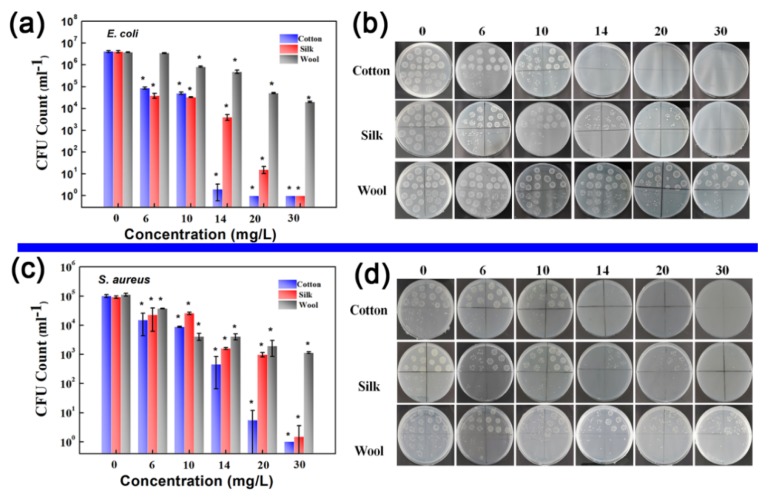
Antibacterial activities of as-prepared Ag, Au, and Pt NP-coated cotton, silk, and wool textiles against *E. coli* and *S. aureus*. (**a**,**c**) CFU content of cotton, silk, and wool textiles after incubation with *E. coli* and *S. aureus* for 24 h: the NP mixture concentrations used to treat the textiles were 6, 10, 14, 20, and 30 mg/L. (**b**,**d**) Corresponding photos of *E. coli* and *S. Aureus* colonies in culture dishes (mean ± SD, * *p* < 0.05).

The developed HBPAA-driven self-assembly strategy has several advantages, including a simple treatment process, clean production, controllable NP content, and wide applicability to mainstream biomass textiles, and thus is beneficial to industrial production. To demonstrate the potential applications of our technology, we coated cotton, silk, and wool textiles with antibacterial Ag, Au, and Pt NP and challenged them with two typical pathogenic bacteria: Gram-negative *E. coli* and Gram-positive *S. aureus*. The antibacterial effects of the treated textiles are shown in Figure 7a–d. The antibacterial rates of treated biomass textiles showed a high positive correlation with NP content. When the NP concentration was increased from 6 to 30 mg/L, the antibacterial activities of all textile samples against *E. coli* and *S. aureus* increased to 99% (Table 2). However, wool fabric showed much lower antibacterial activities against *E. coli* and *S. aureus* than cotton and silk fabrics. Since the crystallinity of wool is only 14%–18% [33], which is much lower than that of cotton (65%–72%) and silk (40%–60%), a large portion of nanoparticles were probably adsorbed into the amorphous region of wool fibers, which restricted the release of antibacterial active substances. Moreover, the NP-treated biomass textiles showed higher antibacterial activity toward *E. coli* than *S. aureus* due to differences in the cell wall architecture of the pathogens. 

## 4. Conclusions

A new approach to prepare single-layered Ag, Au, and Pt nanocrystal ternary-coated cotton, silk, and wool textiles through HBPAA-driven self-assembly was demonstrated. HBPAA is a powerful “paint” that could transform Ag, Au, and Pt NPs into homogeneous NPs with similar chemical nature, e.g., strong chemical affinity to biomass materials. The ternary heterogeneous coating was constructed by the one-step impregnation of biomass textiles in a boiled colloidal solution of Ag, Au, and Pt NPs. FESEM analysis indicated that the Ag, Au, and Pt NPs were randomly monodispersed on the cotton, silk, and wool surfaces and formed a single-layered coating due to strong electrostatic repulsion among positively charged NPs. XPS characterization further showed that HBPAA could be successfully anchored onto biomass surfaces, thereby verifying its strong binding ability to biomass matter. The developed polymer-driven self-assembly strategy represents a simple, clean, NP-content controllable, and widely applicable NP coating technology that may open up a new route for the preparation of multiple nanocoatings on fibers in a green and efficient manner.

## Figures and Tables

**Table 1:** XPS peaks of the treated cotton, silk, and wool textiles.

Sample	Binding Energy (eV)					
	Ag		Au		Pt	
	3*d*_5/2_	3*d*_3/2_	4*f*_7/2_	4*f*_5/2_	4*f*_7/2_	4*f*_5/2_
Cotton	367.8	373.8	83.65	87.36	74.21	71
Silk	367.6	373.6	83.53	87.23	74.2	71.03
Wool	367.65	373.62	83.59	87.36	74.26	70.64
*Bulk metal*	367.9	373.9	83.8	87.45	74.25	70.9

**Table 2:** Antibacterial rates of as-prepared Ag, Au, and Pt NP-coated cotton, silk, and wool textiles.

NP Concentration (mg/L)	Antibacterial Rates (%)					
	Cotton		Silk		Wool	
	*E. coli*	*S. aureus*	*E. coli*	*S. aureus*	*E. coli*	*S. aureus*
6	97.9	85	99.06	75.68	7.59	66.22
10	98.78	91.2	99.2	72.32	78.53	93.87
14	100	99.55	99.9	98.27	87.17	96.3
20	100	~100	~100	98.94	98.66	98.26
30	100	~100	100	~100	99.48	99.33
